# Editorial: Innovations in Imaging for Early Diagnosis and Monitoring for Patients With Gastrointestinal Cancer

**DOI:** 10.3389/fonc.2022.913387

**Published:** 2022-05-27

**Authors:** Stephen J. Pandol, Temel Tirkes, Debiao Li

**Affiliations:** ^1^ Department of Medicine, Cedars-Sinai Medical Center, Los Angeles, CA, United States; ^2^ Department of Radiology and Imaging Sciences, Indiana University School of Medicine, Indianapolis, IN, United States; ^3^ Biomedical Imaging Research Institute, Cedar-Sinai Medical Center, Los Angeles, CA, United States

**Keywords:** cancer, imaging, computerized tomography, magnetic resonance imaging, artificial intelligence

## Overview

Medical imaging is an essential tool for risk assessment, early detection, therapeutic decision making and monitoring of abdominal cancers such as liver, pancreas and luminal gastrointestinal cancers. Various CT, PET, MRI and endoscopic techniques have been developed in recent years having an impact on these critical medical disorders.

We proposed this Research Topic to act as a platform for presenting new methods in imaging that can be developed into applicable clinical tools for a variety of tasks in management of gastrointestinal cancers. We posited that new methods development will play an increasingly important role in several aspects of cancer management with the goal of improving outcomes for patients who are at risk or who have one of the gastrointestinal cancers. Furthermore, we anticipate that although the methods described in the papers in this Research Topic are usually applied to one cancer, lessons learned about a method for an individual cancer will likely be applicable to other cancers. For a summary of the papers in this series, we have grouped methods presented around specific diseases for readers to consider further development for the disease; and/or considering development of the method(s) used in management of another cancer whether in the gastrointestinal tract or elsewhere.

## Pancreatic Diseases

Several papers in this Research Topic addressed methods for *pancreatic diseases*. Distinguishing different forms of pancreatic cancer- pancreatic ductal adenocarcinoma (PDAC) and pancreatic neuroendocrine tumors from each other and inflammatory pancreatitis is often challenging. Two papers in this Research Topic address the differential diagnosis of these diseases using; 1. multiparametric mapping [from T1-weighted imaging (T1WI), T2-weighted imaging (T2WI) and apparent diffusion coefficient (ADC)] in defining tissue characteristics showing that the combination of measures can distinguish between the pancreatic disorders (Wang et al.); and using a radiomics approach using T1WI, T2WI during the arterial and portal phases of contrast-enhanced MRI performed better than clinical evaluation of the imaging data (Deng et al.). Other papers used radiomics approaches combined with clinical information to better identify vascular and lymph node involvement in PDAC and predict outcome for the patient (Chen et al., Gao et al., and Cen et al.). Additional papers used CT and contrast enhanced CT (CE-CT) to glean PDAC characteristics that predict outcome (Zaid et al. and Xu et al.). In one of these papers, (Xu et al.) the authors demonstrated that greater contrast enhancement is associated with better patient outcomes while another paper (Zaid et al.) showed that differences in CT characteristics between the tumor and the surrounding “normal” tissue is also a predictor of outcome. As an example of theranostics, one of the papers in the series demonstrated the feasibility of combining endoscopic optic coherence tomography with brachytherapy for the treatment of early PDAC lesions (Lu et al.). Finally, as an example of developments for future MRI applications for identifying specific molecular signatures in a cancer, a paper in the series shows preclinical studies using a probe for fibronectin (Qiao et al.).

A clinically valuable study (Wang et al.) used clinical and imaging findings for creating a robust algorithm to distinguish between a benign from malignant bulging duodenal papilla (papilla of Vater). The paper is important as early identification of a malignant papilla (a subset of pancreatic cancers) leads to improved outcomes.

In contrast to PDAC tumors of pancreas which have almost universal deadly outcome unless treated, neuroendocrine tumors of the pancreas have much more variable biologic behavior. Thus, developing methods to enhance to ability to distinguish malignant neuroendocrine tumors from those with a more benign clinical course are highly valuable. Two papers in the series address this issue (Klimov et al. and Zhang et al.). One study (Klimov et al.) uses a machine learning algorithm of routine histology of the neuroendocrine tumor to predict risk of metastasis while that other (Zhang et al.) uses machine learning radiomics from CT studies to show association with pathologic grade based on microscopy.

## Colorectal Carcinoma

The series has 5 studies based on methods that can be applied to colorectal carcinoma management (Liu et al., Han et al., Maslova et al., and Wei et al.). These studies show that T2WI images show the best ability to predict extramural venous invasion; (Liu et al.) that the mucin pool content measured by MRI prior to neoadjuvant therapy (NAT) can predict therapy outcome in locally advanced rectal mucinous adenocarcinoma (Cao et al.); that endoscopic ultrasound is highly accurate in distinguishing different stages for colorectal cancer (Han et al.); and that contrast enhanced CT is reliable for predicting responses to chemoradiation of rectal carcinoma allowing a “watch and wait” strategy before applying additional therapy (Maslova et al.). An additional paper demonstrated that expression of the Chloride Channel Accessory 1 (CLCA1) gene may be a candidate diagnostic and prognostic biomarker for CRC (Wei et al.). This last study is a reminder that in the future, multiple measurements including clinical and imaging data but also tissue biomarkers will provide the most advance approaches for early diagnosis and precision application of treatment. A difficult to diagnosis case of intestinal Ewing’s sarcoma (Yang et al.). The report illustrates the complementary roles of different imaging modalities in identifying and treating difficult cases.

## Hepatocellular Carcinoma

Three papers address novel imaging methods for management of hepatocellular carcinoma (HCC)^-^ (Wen et al., Sung et al., and Wu et al.). These studies address the role of radiomics analysis of MRI based imaging in predicting the potential recurrence HCC after treatment with either surgery or radiofrequency ablation (Wen et al.); and the potential use of MRI apparent diffusion coefficient (ADC) mapping to predict responses to hepatic intra-arterial cisplatin chemotherapy (Sung et al.). An thought-provoking report showed the role of a relatively novel MRI imaging technique called amide proton transfer (APT) imaging in predicting the histologic grade of HCC. APT is a form of chemical exchange saturation transfer (CEST) that measures the frequency of transfer of protons between amide groups of proteins and H_2_O (Wu et al.).

## Gastric and Esophageal Carcinoma

Papers addressing gastric cancer show that CT features of the lesion can predict that presence of DNA Mismatch Repair Deficiency (Cao et al.); and that endoscopic molecular imaging with fluorescent probes to surface cancer markers can be used to reveal early gastric cancer (An et al.). Those for esophageal carcinoma demonstrate the predictive capability of contrast-enhanced CT-based radiomic features to distinguish subtle differences in tumor stage for those cancers located at the gastroesophageal junction (Chang et al.); and that response of esophageal squamous cell carcinoma can be predicted from measures of vascular permeability and texture parameters with contrast enhanced MRI (Ji et al.).

## Future Directions

The papers in this Research Topic show by examples the enormous potential of several imaging techniques for diagnosis, outcome prediction and responses to therapeutic interventions. In addition, some papers show that future directions can involve measurements that reflect histology and biologic behavior of cancers and potentially even biochemistry of its constituents by MRI methods such as APT (amide proton transfer) (1) and CEST (chemical exchange saturation transfer) (2). Additional overarching observation is that radiomics and artificial intelligence will be necessary analytic tools accompanying advances in imaging. The application of artificial intelligence analytics will furthermore involve addition of patient clinical data and other potential revealing information including germline and tumor genetics. Finally, as suggested by a Meta-Analysis of studies measuring Volatile Organic Compounds (VOCs) (Xiang et al.) other measures whether volatile or liquid biomarkers will provide further enhancement of diagnostic and predictive algorithms. The stage is set for rapid advancements that will certainly lead to better outcomes for patients.

## Author Contributions

All authors listed have made a substantial, direct, and intellectual contribution to the work and approved it for publication.

## Funding

This study was funded by the United States National Cancer Institute (R01CA260955).

## Conflict of Interest

The authors declare that the research was conducted in the absence of any commercial or financial relationships that could be construed as a potential conflict of interest.

## Publisher’s Note

All claims expressed in this article are solely those of the authors and do not necessarily represent those of their affiliated organizations, or those of the publisher, the editors and the reviewers. Any product that may be evaluated in this article, or claim that may be made by its manufacturer, is not guaranteed or endorsed by the publisher.
